# The Relationship Between Elevated Serum Uric Acid and Risk of Stroke in Adult: An Updated and Dose–Response Meta-Analysis

**DOI:** 10.3389/fneur.2021.674398

**Published:** 2021-08-10

**Authors:** Tianci Qiao, Hongyun Wu, Wei Peng

**Affiliations:** ^1^Graduate School, Shandong University of Traditional Chinese Medicine, Jinan, China; ^2^No.3 Neurology Department, Shandong University of Traditional Chinese Medicine Affiliated Hospital, Jinan, China

**Keywords:** risk factor, meta-analysis, serum uric acid, ischemic stroke, hemorrhagic stroke

## Abstract

**Background:** Uric acid (UA) is proposed as a potential risk factor for stroke in adult, yet the results from published studies are not generally accordant.

**Method:** We included prospective studies that explored the relationship between serum UA (SUA) and strokes. In this study, strokes include ischemic stroke and hemorrhagic stroke, which consists of intracerebral hemorrhage and subarachnoid hemorrhage. The effect-size estimates were expressed as hazard ratio (HR) and 95% confidence interval (CI). Sensitivity and subgroup analyses were performed to assess the robustness of the pooled estimation and potential sources of heterogeneity between studies.

**Results:** We meta-analyzed 19 prospective cohort articles, which involve 37,386 males and 31,163 females. Overall analyses results showed a significant association between a 1 mg/dl increase in high levels of SUA and the risk of total stroke (HR = 1.13; 95% CI: 1.09–1.18; *P* < 0.001), ischemic stroke (HR = 1.15; 95% CI: 1.10–1.21; *P* < 0.001), and hemorrhagic stroke (HR = 1.07; 95% CI: 1.00 to 1.15; *P* = 0.046). No significant difference was found between ischemic stroke and hemorrhagic stroke. In the subgroup analyses, the association of high SUA levels and the risk of total stroke was statistically significant in females (HR = 1.19; 95% CI: 1.12–1.26; *P* < 0.001) and males (HR = 1.11; 95% CI: 1.05–1.17; *P* < 0.001). Coincidentally, the association was also statistically significant for ischemic stroke, both in females (HR = 1.26; 95% CI: 1.17–1.36; *P* < 0.001) and in males (HR = 1.12; 95% CI: 1.06–1.19; *P* < 0.001). However, for hemorrhagic stroke, it was only statistically significant in females (HR = 1.19; 95% CI: 1.04–1.35; *P* = 0.01). Our dose–response research indicated the J-shaped trend between the ascending SUA levels and the higher risk of suffering from a stroke.

**Conclusions:** Our findings indicate that elevated SUA is a significant risk factor for adult stroke, both for ischemic stroke and hemorrhagic stroke, and especially in females.

## Introduction

Stroke is believed to be the second leading cause of death and a major contributor to disability-adjusted life-years (DALYs) lost worldwide (1). According to global statistics, together with ischemic heart disease, strokes account for nearly 15.2 million deaths in 2015 (1). In 2017, intracerebral hemorrhage and ischemic stroke caused 57.9 and 47.8 million DALYs lost, separately (2). Stroke is preventable. Multiple modifiable risk factors, such as hypertension, diabetes mellitus, atrial fibrillation, dyslipidemia, smoking, obesity, lack of physical activity, etc., have been widely observed in the prevention and treatment of stroke (1). However, the number of incidents of stroke, survivors, and stroke-related death, as well as DALYs, are still increasing globally (3). Therefore, a better understanding of more potential risk factors are needed to develop additional preventive strategies for stoke.

Uric acid (UA), one metabolic end product of purine, exists in the form of UA salt with high solubility in organisms. Regularly, serum UA (SUA) levels range from 1.5 to 6.0 mg/dl for women and 2.5 to 7.0 mg/dl for men under a healthy status, which is hard upon the upper limit of UA dissolution in serum (4). Up to date, controversial results regarding the correlation between SUA levels and the incidence of stroke have been reported. It was shown that UA is one of the most essential antioxidants in the blood whose concentration is 10 times greater than that of other antioxidants. UA provides an antioxidant defense against oxidant- and radical-caused damage in humans (5). Researches demonstrated that UA is an antioxidant factor to protect nerves from oxidative damage (6, 7), thereby possibly preventing stroke outcomes. Whereas, many studies found that high SUA levels might be a major risk factor for the onset of stroke (8–11). Zhong et al. explored the association between SUA levels and risk of stroke base on a meta-analysis (12). The study revealed that the elevated SUA levels were significantly related to the modestly increased risk of stroke, and there existed no significant gender differences. Meanwhile, the association between SUA and the risk of each subtype of stroke had been developed by different meta-analyses (13, 14). No studies were conducted to compare the effect of SUA levels on ischemic stroke and hemorrhagic stroke. It is widely accepted that hemorrhagic stroke is ascribed to the rupture of a blood vessel, and ischemic stroke is caused by blockage of an artery; both conditions cause local hypoxia that damages brain tissue. Ischemic stroke accounts for the majority of strokes, yet hemorrhagic stroke is responsible for more deaths and DALYs lost (15). Identifying the role of SUA levels in each type of stroke is vital for subsequent targeted treatment and prevention. In our study, we performed a meta-analysis of prospective studies to detect the association between elevated SUA levels and the risk of stroke and explored the differences between ischemic stroke and hemorrhagic stroke.

## Methods

This meta-analysis was carried out in line with the guidelines of the Preferred Reporting Items for Systematic Reviews and Meta-analyses (PRISMA) statement (16), which is presented in Supplementary Table 1.

### Search Strategy

We finished literature search by looking through PubMed, EMBASE, and Web of Science databases as of December 26, 2020. The following medical nomenclature are considered: (uric acid OR ua OR urate OR hyperuricemia OR hyperuric OR ammonium acid urate [Title/Abstract]) AND (stroke OR cerebrovascular OR apoplexy OR brain vascular accident OR cerebral stroke OR ischemic stroke OR ischaemic stroke OR cryptogenic ischemic stroke OR cryptogenic stroke OR embolism stroke OR intracranial embolism OR intracranial infarction OR cerebral embolism OR cerebral infarction OR brain infarction OR intracranial hemorrhage OR brain hemorrhage OR hemorrhagic stroke OR cerebral hemorrhage [Title/Abstract]). In order to avoid underlying missing points, reference lists of retrieved articles and systematic reviews were scanned.

Two researchers (Tianci Qiao and Hongyun Wu) examined all retrieved articles independently, and they seriously assessed preliminary qualification based on the titles, abstracts, and full texts when necessary.

### Inclusion/Exclusion Criteria

We included the articles when they met the following criteria: (1) the study has a prospective design (prospective cohort or prospective nested case-control study); (2) the study outcomes were stroke, including ischemic stroke and any kinds of hemorrhagic stroke (intracerebral hemorrhage and subarachnoid hemorrhage); (3) enrolled participants were free of stroke at baseline; (4) studies that reported the definition of outcomes in participants with stroke; and (5) hazard ratio (HR) and corresponding 95% confidence interval (CI) of the association between UA and stroke were reported. Articles were excluded if they were reviews, proceedings, letters, case reports, or meta-analyses, or they were not reported in English languages, or the subjects of the studies were not stroke patients, or they were of duplicated publications or studies using overlapping data.

### Data Extraction

Two investigators (Tianci Qiao and Hongyun Wu) excerpted data from each qualified article and imported them into a standardized Excel spreadsheet independently, including name of the first author, year of publication, location where study was conducted, sample size, sex, baseline age, follow-up period, ascertainment of UA and stroke, type of stroke, levels of UA, effect estimation, adjusted confounders, and other traditional risk factors, if available. The disagreements were resolved by reevaluating original articles jointly and, if necessary, by a third author (Wei Peng).

### Statistical Analysis

Stata software version 14.1 for Windows (Stata Corp, College Station, TX, USA) was used to regulate and analyze the data. The random-effects model was employed without considering the magnitude of between-study heterogeneity. Effect size estimates were indicated by HR and its 95% CI. The difference between the two estimates was tested by using *Z*-test as reported by Altman and Bland (17). Generalized least squares regression proposed by Greenland and Longnecker (18) was used to examine the dose–response association for trend estimation of summarized dose–response data. In addition, non-linearity test between SUA levels and risk of stroke was conduct by restricted cubic splines of exposure distribution with three knots (25, 50, and 75th percentiles).

Heterogeneity between studies was assessed by inconsistency index (*I*^2^), which represents the percentage of multiplicity observed between studies whose result is from chance rather than a casual result. A higher *I*^2^ value indicates a higher degree of heterogeneity. If the *I*^2^ value is higher than 50%, significant heterogeneity would be recorded. As for multiple sources of heterogeneity possibly from clinical and methodological fields, plenty of prespecified subgroups were analyzed according to the baseline age, gender, region, follow-up, factor correction, including whether body mass index (BMI) was adjusted, smoking status, hypertension or blood pressure, diabetes mellitus or blood glucose, hyperlipidemia or lipid, or renal factors.

Begg's funnel plots and Egger regression asymmetry tests were used to evaluate the potential publication bias at a significance level of 10%. In addition, the number of theoretically missing studies was estimated by trim and fill methods, respectively. Sensitivity analysis was conducted to test the stability of results.

## Results

### Eligible Studies

**Figure 1:**
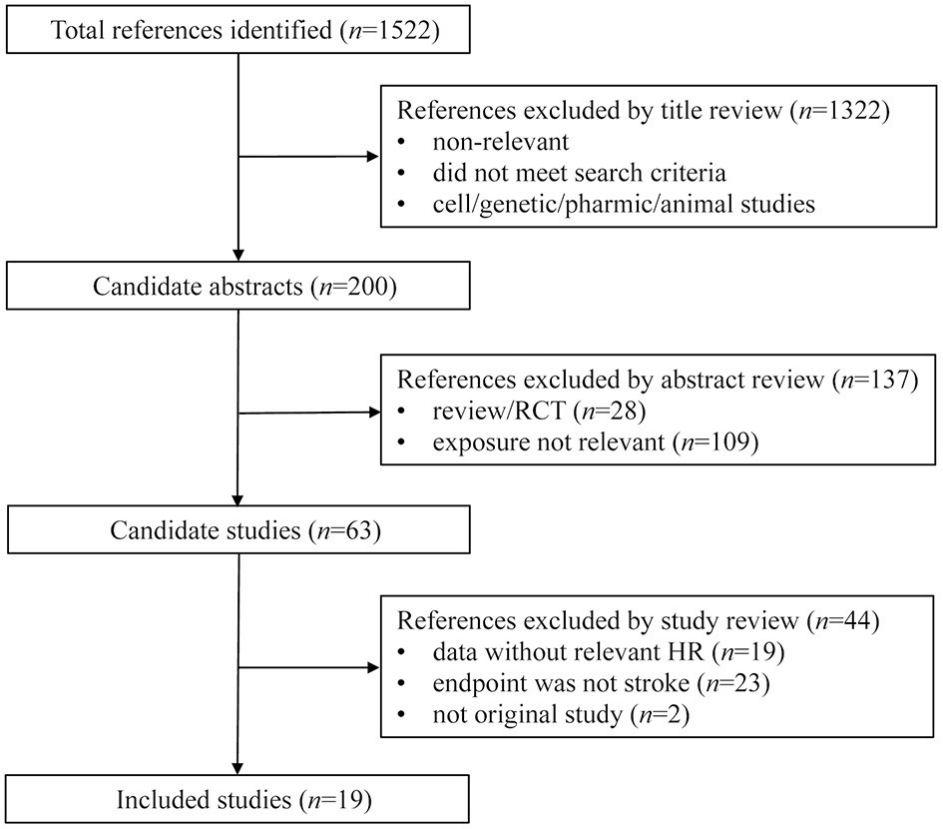
Flow chart of records retrieved, screened and included in this meta-analysis.

A total of 1,522 articles were initially included. After searching the public databases with medical subject terms that were previously defined, there were 19 articles with data on association between SUA and risk of stroke that were eligible for inclusion (11, 19–36), including 37,386 males and 31,163 females in the final analysis. The detailed selection process including specific reasons for exclusion was tabulated in Figure 1.

### Study Characteristics

**Figure 2:**
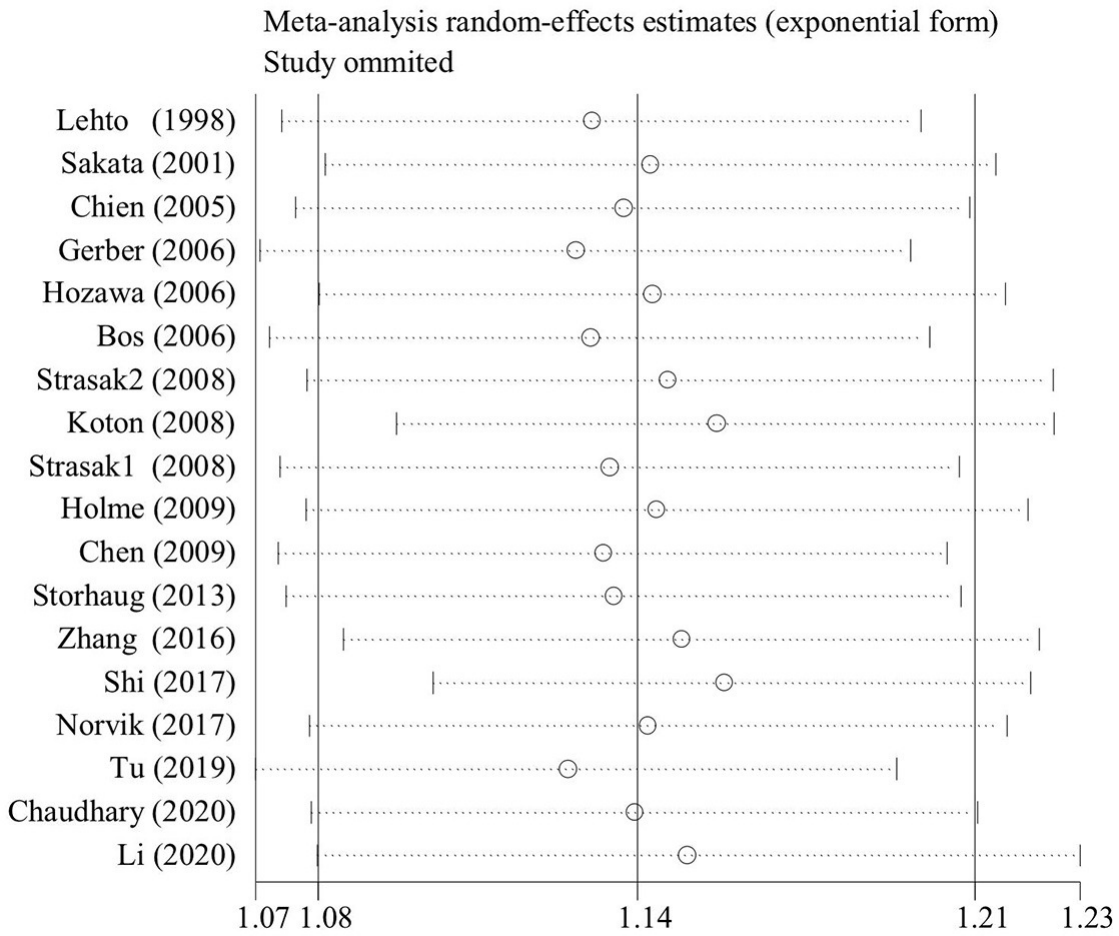
The sensitive plot on the association of uric acid levels and risk of stroke with the exclusion of lower-quality studies.

The baseline characteristics of all cohort studies included in this meta-analysis are displayed in Table 1 and Supplementary Table 1. Only four of 19 qualified articles analyzed the effect of per unit UA increase on stroke (11, 25, 30, 32). Seven articles described the association between different SUA levels and risk of stroke without out separate gender groups (19, 24, 25, 28, 30, 32, 33), and 10 articles specifically reported the effect of different levels of UA on different type of strokes (22, 23, 26–29, 31, 33, 34, 36). Based on geographic regions, all the eligible articles were classified into three categories, namely, America (24, 35), Europe (11, 19, 22, 25–27, 29, 32), and Asia (20, 21, 23, 28, 30, 31, 33, 34, 36). According to sensitive analysis with the exclusion of lower-quality study (30), the outcome was stable (Figure 2).

**Table 1:** Baseline characters of the associations between UA levels and the risk of having stroke.

**Author**	**Year**	**Location**	**Baseline age**	**Follow-up (year)**	**Sample size (*n*)**	**Stroke type**	**Sex**	**Case (*n*)**	**Uric acid levels (μmol/L)**	**HR (95% CI)**
Sakata	2001	Japan	≥30	14	8,172	Total stroke	M	94	297–338	0.84 (0.45–1.59)
Sakata	2001	Japan	≥30	14	8,172	Total stroke	M	94	339–385	0.66 (0.33–1.33)
Sakata	2001	Japan	≥30	14	8,172	Total stroke	M	94	≥386	1.71 (0.92–3.17)
Sakata	2001	Japan	≥30	14	8,172	Total stroke	F	80	214–248	1.40 (0.54–3.63)
Sakata	2001	Japan	≥30	14	8,172	Total stroke	F	80	249–290	0.95 (0.37–2.45)
Sakata	2001	Japan	≥30	14	8,172	Total stroke	F	80	≥291	1.12 (0.46–2.74)
Chien	2005	China	>35	11	3,602	Total stroke	M	155	Per unit	1.13 (0.88–1.46)
Chien	2005	China	>35	11	3,602	Total stroke	F	155	Per unit	1.32 (1.01–1.73)
Bos	2006	Netherlands	≥55	8.4	4,385	Total stroke	M	132	310–375	1.78 (1.16–2.74)
Bos	2006	Netherlands	≥55	8.4	4,385	Total stroke	M	132	≥375	1.41 (0.90–2.23)
Bos	2006	Netherlands	≥55	8.4	4,385	Total stroke	M	132	Per unit	1.15 (0.95–1.38)
Bos	2006	Netherlands	≥55	8.4	4,385	Total stroke	F	249	263–321	1.45 (1.05–2.02)
Bos	2006	Netherlands	≥55	8.4	4,385	Total stroke	F	249	≥321	1.45 (1.05–2.01)
Bos	2006	Netherlands	≥55	8.4	4,385	Total stroke	F	249	Per unit	1.18 (1.05–1.34)
Bos	2006	Netherlands	≥55	8.4	4,385	IS	M	73	310–375	1.57 (0.88–2.79)
Bos	2006	Netherlands	≥55	8.4	4,385	IS	M	73	≥375	1.36 (0.74–2.48)
Bos	2006	Netherlands	≥55	8.4	4,385	IS	M	73	Per unit	1.18 (0.92–1.51)
Bos	2006	Netherlands	≥55	8.4	4,385	IS	F	132	263–321	1.44 (0.91–2.27)
Bos	2006	Netherlands	≥55	8.4	4,385	IS	F	132	≥321	1.68 (1.08–2.62)
Bos	2006	Netherlands	≥55	8.4	4,385	IS	F	132	Per unit	1.26 (1.07–1.49)
Bos	2006	Netherlands	≥55	8.4	4,385	HS	M	16	310–375	1.23 (0.38–4.04)
Bos	2006	Netherlands	≥55	8.4	4,385	HS	M	16	≥375	1.11 (0.32–3.83)
Bos	2006	Netherlands	≥55	8.4	4,385	HS	M	16	Per unit	0.97 (0.55–1.70)
Bos	2006	Netherlands	≥55	8.4	4,385	HS	F	30	263–321	1.22 (0.48–3.10)
Bos	2006	Netherlands	≥55	8.4	4,385	HS	F	30	≥321	1.32 (0.53–3.26)
Bos	2006	Netherlands	≥55	8.4	4,385	HS	F	30	Per unit	1.23 (0.87–1.74)
Gerber	2006	Israel	≥40	23	9,125	Total stroke	M	292	≤238	1.52 (1.04–2.23)
Gerber	2006	Israel	≥40	23	9,125	Total stroke	M	292	238–267	1.46 (1.00–2.12)
Gerber	2006	Israel	≥40	23	9,125	Total stroke	M	292	298–333	1.25 (0.85–1.84)
Gerber	2006	Israel	≥40	23	9,125	Total stroke	M	292	≥333	1.20 (0.81–1.78)
Gerber	2006	Israel	≥40	23	9,125	IS	M	292	≤238	1.34 (0.87–2.05)
Gerber	2006	Israel	≥40	23	9,125	IS	M	292	238–267	1.33 (0.89–2.01)
Gerber	2006	Israel	≥40	23	9,125	IS	M	292	298–333	1.21 (0.81–1.82)
Gerber	2006	Israel	≥40	23	9,125	IS	M	292	≥333	1.15 (0.75–1.74)
Gerber	2006	Israel	≥40	23	9,125	HS	M	292	≤238	3.27 (1.14–9.33)
Gerber	2006	Israel	≥40	23	9,125	HS	M	292	238–267	2.52 (0.87–7.29)
Gerber	2006	Israel	≥40	23	9,125	HS	M	292	298–333	1.55 (0.49–4.89)
Gerber	2006	Israel	≥40	23	9,125	HS	M	292	≥333	1.62 (0.51–5.18)
Hozawa	2006	USA	45–64	12.6	11,263	IS	All	381	286–351	0.86 (0.60–1.23)
Hozawa	2006	USA	45–64	12.6	11,263	IS	All	381	351–411	1.09 (0.79–1.49)
Hozawa	2006	USA	45–64	12.6	11,263	IS	All	381	≥411	1.25 (0.91–1.73)
Hozawa	2006	USA	45–64	12.6	11,263	IS	M	149	286–351	1.01 (0.48–2.13)
Hozawa	2006	USA	45–64	12.6	11,263	IS	M	149	351–411	1.30 (0.67–2.53)
Hozawa	2006	USA	45–64	12.6	11,263	IS	M	149	≥411	1.63 (0.83–3.19)
Hozawa	2006	USA	45–64	12.6	11,263	IS	F	118	286–351	0.85 (0.51–1.41)
Hozawa	2006	USA	45–64	12.6	11,263	IS	F	118	351–411	1.22 (0.75–1.99)
Hozawa	2006	USA	45–64	12.6	11,263	IS	F	118	≥411	1.27 (0.70–2.30)
Strasak1	2008	Austria	62.3	15.2	28,613	Total stroke	F	1,552	220–268	1.25 (0.99–1.57)
Strasak1	2008	Austria	62.3	15.2	28,613	Total stroke	F	1,552	268–322	1.48 (1.18–1.86)
Strasak1	2008	Austria	62.3	15.2	28,613	Total stroke	F	1,552	≥322	1.37 (1.09–1.74)
Strasak1	2008	Austria	62.3	15.2	28,613	Total stroke	F	1,552	Per unit	1.07 (1.01–1.13)
Strasak1	2008	Austria	62.3	15.2	28,613	HS	F	228	220–268	1.14 (0.65–2.01)
Strasak1	2008	Austria	62.3	15.2	28,613	HS	F	228	268–322	1.47 (0.83–2.52)
Strasak1	2008	Austria	62.3	15.2	28,613	HS	F	228	≥322	1.29 (0.71–2.4)
Strasak1	2008	Austria	62.3	15.2	28,613	HS	F	228	Per unit	1.06 (0.91–1.23)
Strasak1	2008	Austria	62.3	15.2	28,613	IS	F	422	220–268	1.33 (0.97–1.83)
Strasak1	2008	Austria	62.3	15.2	28,613	IS	F	422	268–322	1.66 (1.22–2.26)
Strasak1	2008	Austria	62.3	15.2	28,613	IS	F	422	≥322	1.53 (1.11–2.09)
Strasak1	2008	Austria	62.3	15.2	28,613	IS	F	422	Per unit	1.02 (0.91–1.14)
Strasak2	2008	Austria	41.6	13.6	83,683	Total stroke	M	645	273.82–315.48	1.00 (0.76–1.30)
Strasak2	2008	Austria	41.6	13.6	83,683	Total stroke	M	645	315.49–351.19	1.05 (0.80–1.38)
Strasak2	2008	Austria	41.6	13.6	83,683	Total stroke	M	645	351.2–398.81	1.02 (0.78–1.34)
Strasak2	2008	Austria	41.6	13.6	83,683	Total stroke	M	645	>398.81	1.59 (1.23–2.04)
Strasak2	2008	Austria	41.6	13.6	83,683	Total stroke	M	645	Per unit	1.11 (1.05–1.18)
Strasak2	2008	Austria	41.6	13.6	83,683	HS	M	147	273.82–315.48	1.02 (0.60–1.72)
Strasak2	2008	Austria	41.6	13.6	83,683	HS	M	147	315.49–351.19	0.89 (0.51–1.57)
Strasak2	2008	Austria	41.6	13.6	83,683	HS	M	147	351.2–398.81	0.92 (0.53–1.60)
Strasak2	2008	Austria	41.6	13.6	83,683	HS	M	147	>398.81	1.18 (0.70–2.01)
Strasak2	2008	Austria	41.6	13.6	83,683	HS	M	147	Per unit	1.06 (0.93–1.20)
Strasak2	2008	Austria	41.6	13.6	83,683	IS	M	147	273.82–315.48	0.92 (0.52–1.63)
Strasak2	2008	Austria	41.6	13.6	83,683	IS	M	147	315.49–351.19	1.19 (0.68–2.07)
Strasak2	2008	Austria	41.6	13.6	83,683	IS	M	147	351.2–398.81	1.01 (0.57–1.80)
Strasak2	2008	Austria	41.6	13.6	83,683	IS	M	147	>398.81	1.81 (1.07–3.04)
Strasak2	2008	Austria	41.6	13.6	83,683	IS	M	147	Per unit	1.13 (1.01–1.27)
Holme	2009	Sweden	30–85	11.8	417,734	Total stroke	M	9,324	281–319	1.03 (0.97–1.09)
Holme	2009	Sweden	30–85	11.8	417,734	Total stroke	M	9,324	319–362	1.09 (1.02–1.15)
Holme	2009	Sweden	30–85	11.8	417,734	Total stroke	M	9,324	>362	1.26 (1.19–1.34)
Holme	2009	Sweden	30–85	11.8	417,734	Total stroke	F	6,952	208–242	1.05 (0.97–1.15)
Holme	2009	Sweden	30–85	11.8	417,734	Total stroke	F	6,952	242–327	1.16 (1.07–1.26)
Holme	2009	Sweden	30–85	11.8	417,734	Total stroke	F	6,952	>327	1.41 (1.31–1.53)
Holme	2009	Sweden	30–85	11.8	417,734	HS	M	9,324	281–319	0.83 (0.71–0.96)
Holme	2009	Sweden	30–85	11.8	417,734	HS	M	9,324	319–362	0.92 (0.80–1.07)
Holme	2009	Sweden	30–85	11.8	417,734	HS	M	9,324	>362	1.10 (0.96–1.27)
Holme	2009	Sweden	30–85	11.8	417,734	HS	F	6,952	208–242	0.81 (0.64–1.01)
Holme	2009	Sweden	30–85	11.8	417,734	HS	F	6,952	242–327	1.01 (0.82–1.24)
Holme	2009	Sweden	30–85	11.8	417,734	HS	F	6,952	>327	1.13 (0.92–1.37)
Holme	2009	Sweden	30–85	11.8	417,734	IS	M	9,324	281–319	1.08 (1.01–1.16)
Holme	2009	Sweden	30–85	11.8	417,734	IS	M	9,324	319–362	1.10 (1.02–1.18)
Holme	2009	Sweden	30–85	11.8	417,734	IS	M	9,324	>362	1.30 (1.22–1.40)
Holme	2009	Sweden	30–85	11.8	417,734	IS	F	6,952	208–242	1.12 (1.01–1.24)
Holme	2009	Sweden	30–85	11.8	417,734	IS	F	6,952	242–327	1.27 (1.15–1.40)
Holme	2009	Sweden	30–85	11.8	417,734	IS	F	6,952	>327	1.56 (1.42–1.72)
Storhaug	2013	Norway	≥25	12.5	5,700	IS	M	430	Per unit	1.31 (1.14–1.50)
Storhaug	2013	Norway	≥25	12.5	5,700	IS	F	430	Per unit	1.13 (0.94–1.36)
Zhang	2016	Japan	35–89	10	36,313	Total stroke	M	301	279.7–315.4	0.83 (0.58–1.18)
Zhang	2016	Japan	35–89	10	36,313	Total stroke	M	301	315.4–351.1	0.77 (0.52–1.13)
Zhang	2016	Japan	35–89	10	36,313	Total stroke	M	301	351.1–398.7	0.77 (0.52–1.13)
Zhang	2016	Japan	35–89	10	36,313	Total stroke	M	301	398.7–952.2	1.19 (0.84–1.68)
Zhang	2016	Japan	35–89	10	36,313	IS	M	301	279.7–315.4	0.87 (0.54–1.40)
Zhang	2016	Japan	35–89	10	36,313	IS	M	301	315.4–351.1	0.75 (0.45–1.26)
Zhang	2016	Japan	35–89	10	36,313	IS	M	301	351.1–398.7	0.91 (0.55–1.50)
Zhang	2016	Japan	35–89	10	36,313	IS	M	301	398.7–952.2	1.19 (0.75–1.90)
Zhang	2016	Japan	35–89	10	36,313	HS	M	301	279.7–315.4	0.90 (0.46–1.77)
Zhang	2016	Japan	35–89	10	36,313	HS	M	301	315.4–351.1	1.07 (0.54–2.14)
Zhang	2016	Japan	35–89	10	36,313	HS	M	301	351.1–398.7	0.83 (0.41–1.68)
Zhang	2016	Japan	35–89	10	36,313	HS	M	301	398.7–952.2	1.41 (0.75–2.65)
Zhang	2016	Japan	35–89	10	36,313	Total stroke	F	293	202.3–232.1	1.27 (0.90–2.01)
Zhang	2016	Japan	35–89	10	36,313	Total stroke	F	293	232.1–261.8	0.98 (0.62–1.54)
Zhang	2016	Japan	35–89	10	36,313	Total stroke	F	293	261.8–303.5	1.05 (0.67–1.64)
Zhang	2016	Japan	35–89	10	36,313	Total stroke	F	293	303.5–642.7	1.46 (0.98–2.19)
Zhang	2016	Japan	35–89	10	36,313	IS	F	293	202.3–232.1	1.42 (0.74–2.74)
Zhang	2016	Japan	35–89	10	36,313	IS	F	293	232.1–261.8	0.80 (0.40–1.61)
Zhang	2016	Japan	35–89	10	36,313	IS	F	293	261.8–303.5	1.22 (0.65–2.30)
Zhang	2016	Japan	35–89	10	36,313	IS	F	293	303.5–642.7	1.35 (0.75–2.44)
Zhang	2016	Japan	35–89	10	36,313	HS	F	293	202.3–232.1	1.41 (0.64–3.13)
Zhang	2016	Japan	35–89	10	36,313	HS	F	293	232.1–261.8	1.33 (0.63–2.80)
Zhang	2016	Japan	35–89	10	36,313	HS	F	293	261.8–303.5	1.09 (0.48–2.43)
Zhang	2016	Japan	35–89	10	36,313	HS	F	293	303.5–642.7	1.54 (0.76–3.10)
Shi	2017	China	45–75	4.5	20,577	Total stroke	All	632	M: 279.7–327.3 F: 226.1–261.8	0.90 (0.72–1.13)
Shi	2017	China	45–75	4.5	20,577	Total stroke	All	632	M: 327.3–380.8 F: 261.8–309.5	0.90 (0.71–1.13)
Shi	2017	China	45–75	4.5	20,577	Total stroke	All	632	M: ≥380.8 F: ≥309.5	0.87 (0.69–1.11)
Shi	2017	China	45–75	4.5	20,577	IS	All	632	M: 279.7–327.3 F: 226.1–261.8	1.01 (0.78–1.30)
Shi	2017	China	45–75	4.5	20,577	IS	All	632	M: 327.3–380.8 F: 261.8–309.5	0.93 (0.71–1.20)
Shi	2017	China	45–75	4.5	20,577	IS	All	632	M: ≥380.8 F: ≥309.5	0.95 (0.73–1.25)
Shi	2017	China	45–75	4.5	20,577	HS	All	632	M: 279.7–327.3 F: 226.1–261.8	0.56 (0.32–0.97)
Shi	2017	China	45–75	4.5	20,577	HS	All	632	M: 327.3–380.8 F: 261.8–309.5	0.86 (0.52–1.41)
Shi	2017	China	45–75	4.5	20,577	HS	All	632	M: ≥380.8 F: ≥309.5	0.67 (0.38–1.16)
Shi	2017	China	45–75	4.5	20,577	Total stroke	M	300	279.7–327.3	0.86 (0.62–1.19)
Shi	2017	China	45–75	4.5	20,577	Total stroke	M	300	327.3–380.8	0.91 (0.66–1.27)
Shi	2017	China	45–75	4.5	20,577	Total stroke	M	300	≥380.8	0.80 (0.56–1.15)
Shi	2017	China	45–75	4.5	20,577	Total stroke	F	332	226.1–261.8	0.95 (0.69–1.31)
Shi	2017	China	45–75	4.5	20,577	Total stroke	F	332	261.8–309.5	0.90 (0.65–1.24)
Shi	2017	China	45–75	4.5	20,577	Total stroke	F	332	≥309.5	0.95 (0.68–1.32)
Tu	2019	China	≥65	3	3,243	Total stroke	M	1,309	273.7–309.5	1.10 (1.06–2.55)
Tu	2019	China	≥65	3	3,243	Total stroke	M	1,309	309.5–374.9	1.18 (1.07–2.17)
Tu	2019	China	≥65	3	3,243	Total stroke	M	1,309	≥374.9	2.09 (1.40–4.28)
Tu	2019	China	≥65	3	3,243	IS	M	1,309	273.7–309.5	1.09 (1.05–3.35)
Tu	2019	China	≥65	3	3,243	IS	M	1,309	309.5–374.9	1.13 (1.07–3.37)
Tu	2019	China	≥65	3	3,243	IS	M	1,309	≥374.9	1.69 (1.24–4.80)
Tu	2019	China	≥65	3	3,243	HS	M	1,309	273.7–309.5	1.09 (1.05–3.35)
Tu	2019	China	≥65	3	3,243	HS	M	1,309	309.5–374.9	1.13 (1.07–3.37)
Tu	2019	China	≥65	3	3,243	HS	M	1,309	≥374.9	1.69 (1.24–4.80)
Tu	2019	China	≥65	3	3,243	Total stroke	F	1,309	273.7–309.5	1.15 (1.06–2.39)
Tu	2019	China	≥65	3	3,243	Total stroke	F	1,309	309.5–374.9	1.18 (1.12–2.53)
Tu	2019	China	≥65	3	3,243	Total stroke	F	1,309	≥374.9	2.55 (1.28–5.44)
Tu	2019	China	≥65	3	3,243	IS	F	1,309	273.7–309.5	1.15 (1.06–2.39)
Tu	2019	China	≥65	3	3,243	IS	F	1,309	309.5–374.9	1.18 (1.12–2.53)
Tu	2019	China	≥65	3	3,243	IS	F	1,309	≥374.9	1.49 (1.18–4.24)
Tu	2019	China	≥65	3	3,243	HS	F	1,309	273.7–309.5	2.84 (1.33–6.93)
Tu	2019	China	≥65	3	3,243	HS	F	1,309	309.5–374.9	3.37 (1.55–8.82)
Tu	2019	China	≥65	3	3,243	HS	F	1,309	≥374.9	5.85 (1.99–9.81)
Chaudhary	2020	USA	≥45	4	30,239	Total stroke	M	430	357–404.7	2.11 (1.29–3.45)
Chaudhary	2020	USA	≥45	4	30,239	Total stroke	M	430	≥404.7	1.14 (0.75–1.73)
Chaudhary	2020	USA	≥45	4	30,239	Total stroke	F	389	357–404.7	0.78 (0.46–1.34)
Chaudhary	2020	USA	≥45	4	30,239	Total stroke	F	389	≥404.7	1.04 (0.62–1.73)
Li	2020	Japan	40–79	23.1	13,420	Total stroke	M	488	279.7–321.4	1.03 (0.78–1.36)
Li	2020	Japan	40–79	23.1	13,420	Total stroke	M	488	321.4–357.1	0.95 (0.71–1.27)
Li	2020	Japan	40–79	23.1	13,420	Total stroke	M	488	357.1–398.7	1.10 (0.82–1.48)
Li	2020	Japan	40–79	23.1	13,420	Total stroke	M	488	398.7–666.5	1.02 (0.74–1.35)
Li	2020	Japan	40–79	23.1	13,420	Total stroke	M	488	Per unit	1.02 (0.92–1.13)
Li	2020	Japan	40–79	23.1	13,420	HS	M	488	279.7–321,4	1.06 (0.57–1.98)
Li	2020	Japan	40–79	23.1	13,420	HS	M	488	321.4–357.1	1.23 (0.66–2.29)
Li	2020	Japan	40–79	23.1	13,420	HS	M	488	357.1–398.7	1.26 (0.67–2.41)
Li	2020	Japan	40–79	23.1	13,420	HS	M	488	398.7–666.5	0.83 (0.40–1.72)
Li	2020	Japan	40–79	23.1	13,420	HS	M	488	Per unit	0.95 (0.75–1.19)
Li	2020	Japan	40–79	23.1	13,420	IS	M	488	279.7–321,4	1.04 (0.74–1.45)
Li	2020	Japan	40–79	23.1	13,420	IS	M	488	321.4–357.1	0.89 (0.63–1.26)
Li	2020	Japan	40–79	23.1	13,420	IS	M	488	357.1–398.7	1.01 (0.71–1.44)
Li	2020	Japan	40–79	23.1	13,420	IS	M	488	398.7–666.5	1.01 (0.70–1.41)
Li	2020	Japan	40–79	23.1	13,420	IS	M	488	Per unit	1.02 (0.91–1.15)
Li	2020	Japan	40–79	23.1	13,420	Total stroke	F	530	214.2–244	1.02 (0.74–1.40)
Li	2020	Japan	40–79	23.1	13,420	Total stroke	F	530	244–273.7	1.20 (0.89–1.63)
Li	2020	Japan	40–79	23.1	13,420	Total stroke	F	530	273.7–309.5	1.15 (0.84–1.56)
Li	2020	Japan	40–79	23.1	13,420	Total stroke	F	530	309.5–613	1.45 (1.07–1.96)
Li	2020	Japan	40–79	23.1	13,420	Total stroke	F	530	Per unit	1.12 (1.03–1.22)
Li	2020	Japan	40–79	23.1	13,420	HS	F	530	214.2–244	0.64 (0.32–1.25)
Li	2020	Japan	40–79	23.1	13,420	HS	F	530	244–273.7	0.86 (0.47–1.59)
Li	2020	Japan	40–79	23.1	13,420	HS	F	530	273.7–309.5	1.22 (0.68–2.18)
Li	2020	Japan	40–79	23.1	13,420	HS	F	530	309.5–613	1.20 (0.65–2.20)
Li	2020	Japan	40–79	23.1	13,420	HS	F	530	Per unit	1.19 (0.99–1.42)
Li	2020	Japan	40–79	23.1	13,420	IS	F	530	214.2–244	1.33 (0.88–2.02)
Li	2020	Japan	40–79	23.1	13,420	IS	F	530	244–273.7	1.52 (1.02–2.26)
Li	2020	Japan	40–79	23.1	13,420	IS	F	530	273.7–309.5	1.12 (0.73–1.72)
Li	2020	Japan	40–79	23.1	13,420	IS	F	530	309.5–613	1.61 (1.07–2.41)
Li	2020	Japan	40–79	23.1	13,420	IS	F	530	Per unit	1.06 (0.95–1.18)
Norvik	2017	Norway	55–74	19	2,940	IS	All	271	Per unit	1.13 (1.02–1.25)
Chen	2011	China	19–85	1.5	226	IS	All	43	Per unit	1.01 (0.99–1.01)
Chen	2009	China	≥35	8.2	5,427	IS	All	344	Per unit	1.35 (1.04–1.76)
Chen	2009	China	≥35	8.2	5,427	IS	M	344	>416.6	1.14 (0.83–1.57)
Chen	2009	China	≥35	8.2	5,427	IS	F	344	>416.6	1.83 (1.17–2.87)
Chen	2009	China	≥35	8.2	5,427	HS	All	200	Per unit	1.18 (0.83–1.67)
Chen	2009	China	≥35	8.2	5,427	HS	M	200	>416.6	1.18 (0.76–1.83)
Chen	2009	China	≥35	8.2	5,427	HS	F	200	>416.6	1.01 (0.55–1.88)
Koton	2008	UK	45–85	3.8	2,131	IS	All	259	Per unit	0.94 (0.83–1.06)
Koton	2008	UK	45–85	3.8	2,131	IS	M	259	Per unit	0.90 (0.78–1.04)
Koton	2008	UK	45–85	3.8	2,131	IS	F	259	Per unit	1.07 (0.83–1.38)
Lehto	1998	Finland	45–64	7	1,017	Total stroke	All	114	>295	1.91 (1.24–2.94)

*HR, hazard ratio; 95% CI, 95% confidence interval; IS, Ischemic Stroke; HS, Hemorrhagic Stroke; UA: uric acid; UK, the united kingdom; USA, the United States; M, male; F, female; All, both male and female*.

### Quality Assessment

The Newcastle–Ottawa Scale (NOS) tool was used to assess the quality of the cohort studies, shown in Table 2, with the total scores ranging from 5 to 9 in this meta-analysis.

**Table 2:** The Newcastle-Ottawa Scale (NOS) for assessing the quality of cohort studies.

**First author**	**Sakata**	**Jee**	**Chien**	**Bos**	**Gerber**	**Hozawa**	**Strasak1**	**Strasak2**	**Holme**	**Storhaug**	**Zhang**	**Shi**	**Tu**	**Chaudhary**	**Li**	**Norvik**	**Chen**	**Koton**	**Lehto**
**Year**	**2001**	**2004**	**2005**	**2006**	**2006**	**2006**	**2008**	**2008**	**2009**	**2013**	**2016**	**2017**	**2019**	**2020**	**2020**	**2017**	**2011**	**2008**	**1998**
1. Representativeness of the exposed cohort	1	1	0	0	0	0	1	1	1	0	1	1	0	1	1	1	0	1	1
2. Selection of the non-exposed cohort	1	1	1	1	1	1	1	1	1	1	1	1	1	1	1	1	1	1	1
3. Ascertainment of exposure	1	0	1	1	1	1	1	1	1	1	1	1	1	1	1	1	1	1	1
4. Demonstration that outcome of interest was not present at start study	1	1	1	1	1	1	1	1	1	1	1	1	1	1	1	1	1	1	1
5. Control for important cohort	1	1	1	0	1	1	1	1	1	1	1	1	1	1	1	1	1	1	1
6. Additional factors	1	0	0	0	0	1	1	1	0	1	0	0	1	1	0	0	0	1	0
7. Assessment of outcome	1	1	1	1	1	1	1	1	1	1	1	1	1	1	1	1	1	0	1
8. Follow up	1	1	1	1	1	1	1	1	1	1	1	0	0	0	1	1	0	1	1
9. Adequacy of follow up of cohorts	1	1	1	1	0	1	1	1	0	0	0	0	0	1	0	0	0	0	0
Score	9	7	7	6	6	8	9	9	7	7	7	6	6	8	7	7	5	7	7

### Overall Analyses

After pooling the results of all eligible prospective cohorts together (Table 3), there was a statistically significant association between SUA levels and the risk of total stroke (HR = 1.13; 95% CI: 1.09–1.18; *P* < 0.001), ischemic stroke (HR = 1.15; 95% CI: 1.10–1.21; *P* < 0.001), and hemorrhagic stroke (HR = 1.07; 95% CI: 1.00–1.15; *P* = 0.046) (Table 3). This association was obscured by significant between-study heterogeneity, with the corresponding *I*^2^ of 59.0, 77.0, and 33.7%. No obvious distinction had been found between ischemic stroke and hemorrhagic stroke (two-sample *Z*-test *P* = 0.095).

**Table 3:** Overall and subgroup analyses of the association between UA levels and the risk of stroke.

**Groups**	**Studies (*n*)**	**Total**		**IS**		**HS**	
		**HR (95% CI); *P***	***I*^**2**^**	**HR (95% CI); *P***	***I*^**2**^**	**HR (95% CI); *P***	***I*^**2**^**
**Overall analysis**							
	13/14/11	1.13 (1.09–1.18); <0.001	59.0%	1.15 (1.10–1.21); <0.001	77.0%	1.07 (1.00–1.15); 0.046	33.7%
**Subgroup analysis**							
**By gender**							
Female	10/10/7	1.19 (1.12–1.26); <0.001	55.1%	1.26 (1.17–1.36); <0.001	58.6%	1.19 (1.04–1.35); 0.01	49.5%
Male	11/12/8	1.11 (1.05–1.17); <0.001	56.9%	1.12 (1.06–1.19); <0.001	38.1%	1.01 (0.95–1.07); 0.81	0.0%
All	2/5/2	1.02 (0.79–1.31); 0.89	72.8%	1.02 (0.97–1.10); 0.38	40.3%	0.82 (0.58–1.16); 0.27	51.5%
**By location**							
Asia	7/6/6	1.06 (1.01–1.13); 0.03	25.5%	1.08 (1.02–1.14); 0.01	19.6%	1.17 (1.03–1.34); 0.02	41.2%
Europe	5/8/4	1.20 (1.13–1.27); <0.001	77.8%	1.19 (1.12–1.27); <0.001	75.5%	1.01 (0.95–1.07); 0.76	1.7%
America	1/1/NA	1.13 (1.10–1.18); 0.39	NA	1.10 (0.95–1.28); 0.19	NA	NA	NA
**By follow up years**							
(0, 10)	5/5/4	1.13 (1.02–1.25); 0.02	56.4%	1.10 (1.02–1.19); 0.01	50.3%	1.24 (0.99–1.54); 0.06	57.4%
(10, 20)	6/7/4	1.15 (1.09–1.21); <0.001	69.7%	1.19 (1.12–1.26); <0.001	60.9%	1.10 (0.96–1.07); 0.75	2.7%
(20, 30)	2/2/2	1.13 (1.09–1.18); <0.001	0.2%	1.15 (1.10–1.21); 0.02	0.0%	1.11 (0.98–1.26); 0.11	4.6%
**By age**							
(20, 40)	4/6/3	1.12 (1.04–1.21); <0.001	72.6%	1.18 (1.08–1.30); <0.001	91.1%	1.00 (0.92–1.08); 0.91	16.6%
(40, 50)	6/6/4	1.08 (1.02–1.14); 0.01	42.2%	1.05 (1.00–1.10); <0.001	3.7%	1.04 (0.94–1.14); 0.45	9.9%
(50, 90)	3/4/3	1.28 (1.17–1.40); <0.001	52.6%	1.23 (1.14–1.34); 0.04	25.9%	1.40 (1.14–1.72); <0.001	49.5%
**By stroke severity**							
Fatal	7/8/6	1.17 (1.10– 1.25); <0.001	37.7%	1.20 (1.13– 1.27); <0.001	12.0%	1.24 (1.10– 1.39); <0.001	31.7%
Non-fatal	5/6/3	1.16 (1.10– 1.23); <0.001	70.6%	1.14 (1.07– 1.22); <0.001	84.8%	1.00 (0.94– 1.07); 0.98	10.1%
**Adjusted body mass index (BMI)**							
Yes	11/12/8	1.11 (1.06–1.16); <0.001	40.3%	1.11 (1.07–1.17); <0.001	24.6%	1.31 (1.03–1.24); 0.01	29.5%
No	2/3/2	1.20 (1.11–1.18); <0.001	83.2%	1.23 (1.11–1.37); <0.001	94.0%	1.07 (1.00–1.15); 0.68	27.5%
**Adjusted smoking status**							
Yes	9/11/7	1.10 (1.04–1.17); <0.001	43.6%	1.12 (1.07–1.19); <0.001	27.5%	1.18 (1.05–1.33); 0.01	39.7%
No	4/4/3	1.17 (1.10–1.24); <0.001	73.3%	1.18 (1.09–1.28); <0.001	89.7%	1.00 (0.94–1.07); 0.98	10.1%
**Adjusted hypertension or blood pressure**							
Yes	10/14/9	1.12 (1.07–1.16); <0.001	61.8%	1.14 (1.09–1.20); <0.001	77.5%	1.07 (1.00–1.16); 0.07	40.0%
No	3/1/1	1.29 (1.14–1.46); <0.001	24.8%	1.30 (1.15–1.46); <0.001	0.0%	1.17 (0.91–1.52); 0.23	0.0%
**Adjusted diabetes mellitus or blood glucose**							
Yes	10/10/7	1.12 (1.07–1.17); <0.001	63.7%	1.15 (1.09–1.21); <0.001	81.7%	1.02 (0.96–1.09); 0.58	20.2%
No	3/4/3	1.20 (1.08–1.32); <0.001	38.5%	1.18 (1.10–1.26); <0.001	0.0%	1.37 (1.13–1.65); <0.001	30.9%
**Adjusted hyperlipidemia or lipid**							
Yes	12/13/8	1.12 (1.07–1.17); <0.001	60.0%	1.14 (1.09–1.19); <0.001	56.5%	1.07 (0.99–1.16); 0.09	43.1%
No	1/2/2	1.27 (1.13–1.41); <0.001	17.5%	1.28 (1.10–1.50); <0.001	73.1	1.16 (0.97–1.39); 0.10	0.0%
**Adjusted renal factors**							
Yes	3/3/8	0.99 (0.90–1.09); 0.87	62.7%	1.14 (1.04–1.25); <0.001	8.0%	1.03 (0.99–1.08); 0.15	79.5%
No	10/12/2	1.17 (1.12–1.22); <0.001	23.3%	1.16 (1.10–1.22); <0.001	79.1%	1.41 (0.88–2.26); 0.16	0.0%

*HR, hazard ratio; 95% CI, 95% confidence interval; IS, Ischemic Stroke; HS, Hemorrhagic Stroke; UA: uric acid; BMI: body mass index; NA, not available*.

### Publication Bias

**Figure 3:**
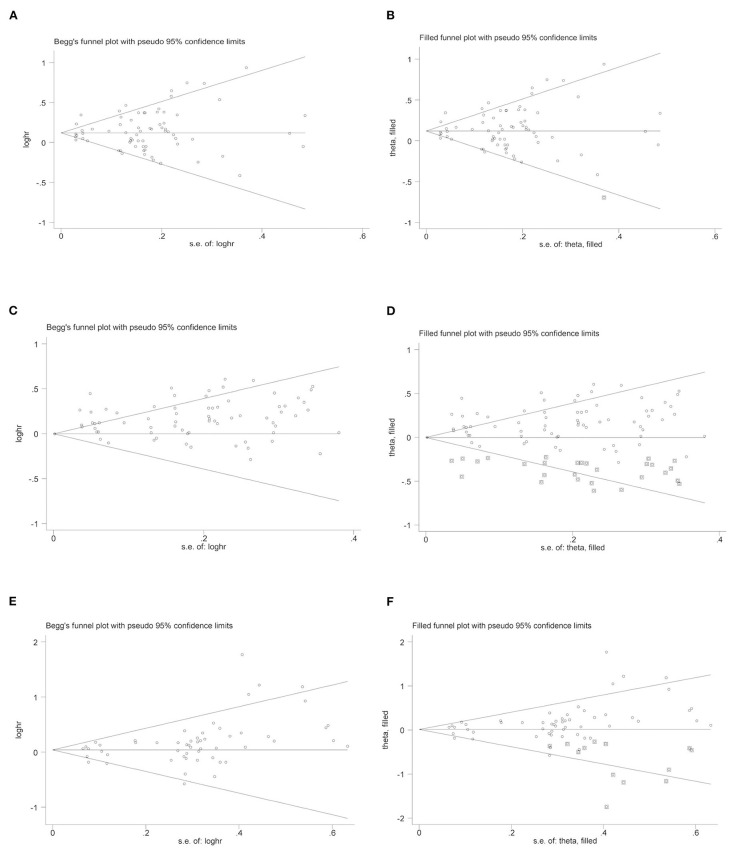
Begg's and filled funnel plots on the association of uric acid levels and risk of stroke. **(A)** Begg's funnel plot and **(B)** Filled funnel plots: UA levels and total stroke. **(C)** Begg's funnel plot and **(D)** Filled funnel plots: UA levels and ischemic stroke. **(E)** Begg's funnel plot and **(F)** Filled funnel plots: UA levels and hemorrhagic stroke.

Begg's funnel plot was used to assess publication bias for the association between SUA levels and risk of stroke, and all of them seemed symmetrical, shown in Figure 3. As exposed by the Egger's test, there were strong evidence of publication bias for total stroke (*P* = 0.00), ischemic stroke (*P* = 0.00), and hemorrhagic stroke (*P* = 0.05). Further filled funnel plots showed that there was one potentially missing study in total stroke, 28 missing studies in ischemic stroke, and 13 missing studies in hemorrhagic stroke due to the publication bias to have a symmetrical plot.

### Subgroup Analyses

A sequence of subgroup analyses was conducted to investigate the possible causes of between-study heterogeneity for SUA levels and risk of stroke (Table 3). By gender, the association of SUA levels and risk of total stroke was statistically significant in both women (HR = 1.19; 95% CI: 1.12–1.26; *P* < 0.001) and men (HR = 1.11; 95% CI: 1.05–1.17; *P* < 0.001) (two-sample *Z*-test *P* = 0.088). It was also statistically significant for ischemic stroke in women (HR = 1.26; 95% CI: 1.17–1.36; *P* < 0.001) and men (HR = 1.12; 95% CI: 1.06–1.19; *P* < 0.001) (two-sample *Z*-test *P* = 0.015). The association of SUA levels and risk of hemorrhagic stroke was statistically significant in women (HR = 1.19; 95% CI: 1.04–1.35; *P* = 0.01), but not in men (HR = 1.01; 95% CI: 0.95–1.07; *P* = 0.81) (two-sample *Z*-test *P* = 0.025).

By geographic locations, in Asia, there was a statistically significant in association between SUA levels and risk of total stroke (HR = 1.06; 95% CI: 1.01–1.13; *P* = 0.03), as well as ischemic stroke (HR = 1.08; 95% CI: 1.02–1.14; *P* = 0.01) and hemorrhagic stroke (HR = 1.17; 95% CI: 1.03–1.34; *P* = 0.02). In Europe, however, there was only statistically significant association for SUA levels and risk of total stroke (HR = 1.20; 95% CI: 1.13–1.27; *P* < 0.001) and ischemic stroke (HR = 1.19; 95% CI: 1.12–1.27; *P* < 0.001).

By follow-up years, in sector of (0, 10) years, significance was observed for association of the SUA levels and risk of total stroke (HR = 1.13; 95% CI: 1.02–1.25; *P* = 0.02) and ischemic stroke (HR = 1.10; 95% CI: 1.02–1.19; *P* = 0.01). For (10, 20) years, total stroke (HR = 1.15; 95% CI: 1.09–1.21; *P* < 0.001) and ischemic stroke (HR = 1.19; 95% CI: 1.12–1.26; *P* < 0.001) were observed to be statistically related to a high level of SUA. While for (20, 30) years as well, the association of the SUA levels and risk of total stroke (HR = 1.13; 95% CI: 1.09–1.18; *P* < 0.001) and ischemic stroke (HR = 1.15; 95% CI: 1.10–1.21; *P* = 0.02) was statistically significant.

By age, total stroke was significantly associated with SUA levels in all subgroups [(20, 40) years: HR = 1.12; 95% CI: 1.04–1.21; *P* < 0.001, (40, 50) years: HR = 1.08; 95% CI: 1.02–1.14; *P* = 0.01, and (50, 90) years: HR = 1.28; 95% CI: 1.17–1.40; *P* < 0.001]. Similarly, for ischemic stroke, statistically significance was observed [(20, 40) years: HR = 1.18; 95% CI: 1.08–1.30; *P* < 0.001, (40, 50) years: HR = 1.05; 95% CI: 1.00–1.10; *P* < 0.001, and (50, 90) years: HR = 1.23; 95% CI: 1.14–1.34; *P* = 0.04]. While for hemorrhagic stroke, only marginal significance was observed among age group of 50–90 years (HR = 1.23; 95% CI: 1.14–1.34; *P* = 0.04).

By the stratification for stroke severity, we classified the severity of a stroke as fatal and non-fatal, and we found high SUA levels were significantly associated with both fatal and non-fatal stroke (fatal stroke: HR = 1.17; 95% CI: 1.10–1.25; *P* < 0.001, non-fatal stroke: HR = 1.16; 95% CI: 1.16–1.23; *P* < 0.001). The same trend was absorbed in ischemic stroke (fatal stroke: HR = 1.20; 95% CI: 1.13–1.27; *P* < 0.001, non-fatal stroke: HR = 1.14; 95% CI: 1.07–1.22; *P* < 0.001) and hemorrhagic stroke (fatal stroke: HR = 1.24; 95% CI: 1.10–1.39; *P* < 0.001, non-fatal stroke: HR = 1.00; 95% CI: 0.94–1.07; *P* = 0.98).

It should also be noticed that the significantly positive associations between SUA levels and risk of stroke that remained in subgroups had been found, which adjusted for potential confounders, including BMI, smoking status, hypertension, diabetes mellitus, hyperlipidemia, and renal factors.

### Dose–Response Analyses

**Figure 4:**
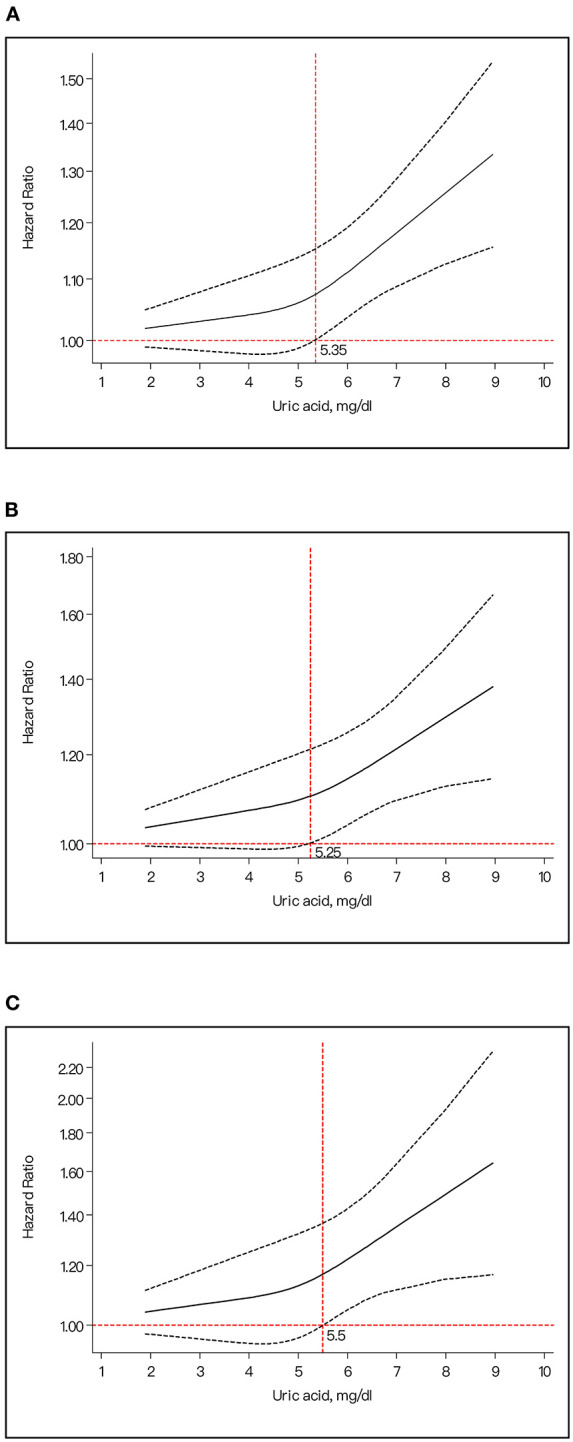
The dose-response plot for the association of uric acid levels and risk of stroke. **(A)** Uric acid levels and risk of total stroke. **(B)** Uric acid levels and risk of ischemic stroke. **(C)** Uric acid levels and risk of hemorrhagic stroke.

Our dose–response research indicated the *J*-shaped trend between the ascending SUA levels and the higher risk of suffering from stroke. In the dose–response analysis on total stroke, the risk of stroke obviously increased with the higher UA concentration. When the SUA reached 5.35 mg/dl, it started to become statistically significant (Figure 4A). The same pattern was also found in ischemic stroke (the dividing value was 5.25 mg/dl) (Figure 4B) and hemorrhagic stroke (5.5 mg/dl) (Figure 4C).

**Figure 5:**
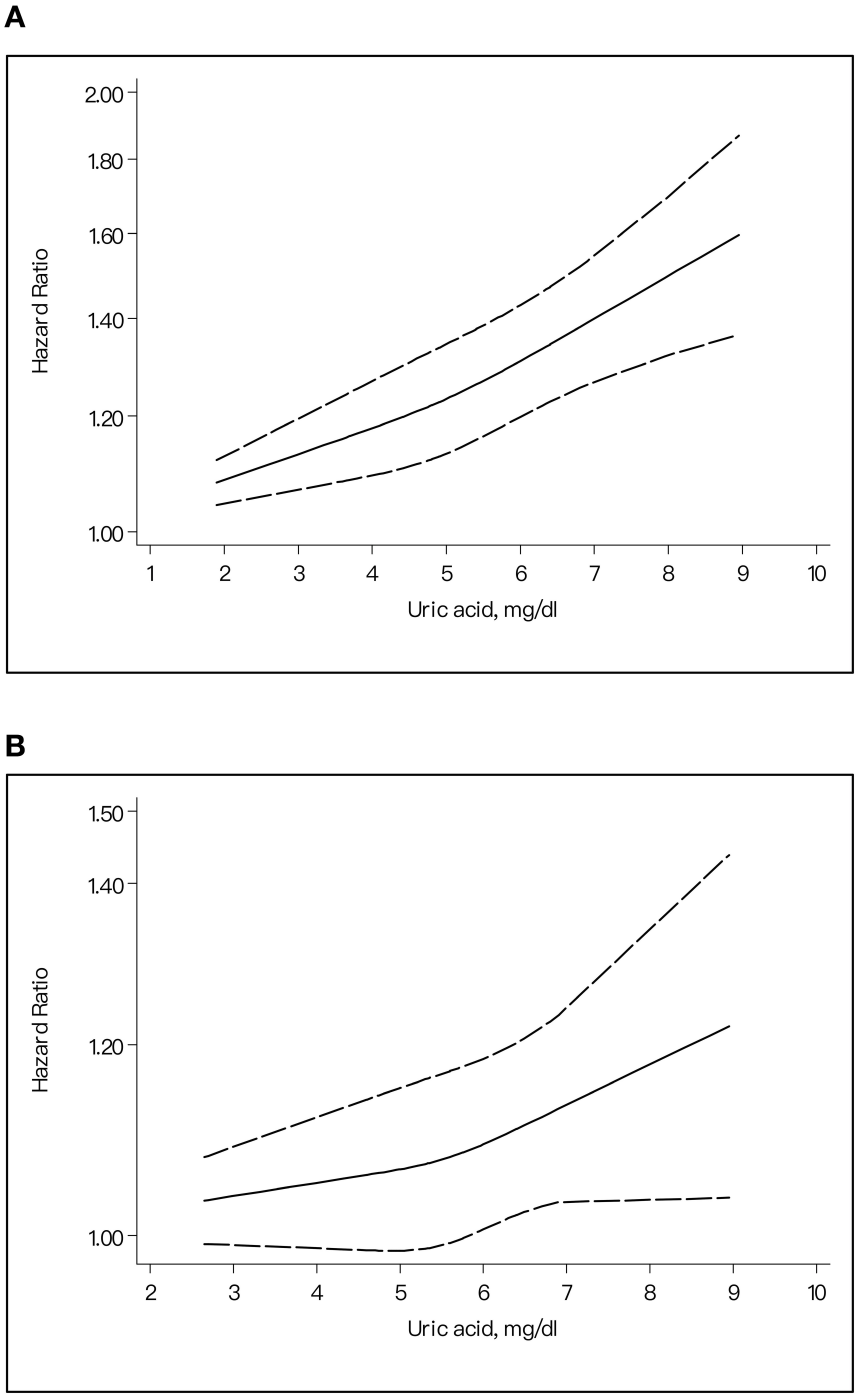
The dose-response plot on the association of uric acid levels and risk of stroke for different gender. **(A)** Female. **(B)** Male.

In our dose–response dichotomized by gender, it indicated a *J*-shaped trend between the ascending SUA levels and the higher risk of stroke for males (*p* for non-linear trend = 0.39) (Figure 5A) and a liner trend (*p* for non-linear trend = 0.32) for females (Figure 5B).

## Discussion

To the best of our knowledge, this is to date the most panoptic meta-analysis that has investigated the association between SUA levels and risk for stroke. The key findings of this study are that elevated SUA is a significant risk factor for adult stroke, both for ischemic stroke and hemorrhagic stroke, and the risk is more evident in females than that in males. Our sensitivity analyses and subgroup analyses also revealed that the relationship between SUA and stroke was robust and not affected by multifactor correction. Moreover, dose–response analysis presented the *J*-shaped trend between the ascending SUA levels and the higher risk of stroke. However, no obvious distinction was found between ischemic stroke and hemorrhagic stroke. More importantly, we found high SUA levels were significantly associated with an increased risk of fatal stroke. Our findings highlight the prominence and the necessity of closely regulating SUA, especially for elderly females, who have a high risk of suffering from cerebrovascular disease.

Several systematic reviews and meta-analyses have evaluated the impact of high SUA on the onset of stroke. Pooling the results of 13 prospective studies by Zhong et al. (12) showed that elevated serum SUA levels were significantly associated with modestly increased risk of stroke and have similar adverse effects on both sexes, whereas further subsidiary analyses by different types of stroke were lacking. Meanwhile, limited seven studies that involved SUA and the risk of stroke in males and seven studies in females had been included in Zhong et al.s' study. Researchers raised that if 10 or fewer studies are pooled in a meta-analysis, the possibility/capacity to detect statistical significance is low (37). At the same time, the study mixed risk ratio (RR) and HR as effect-size estimates for analysis, which is inaccurate and may affect the conclusions. Our work that was based on high-quality cohort studies have avoided these problems effectively and found the same significant relationship.

The concentration of UA is the key point of the mechanisms underlying the association of UA with development of stroke. As one of the most abundant antioxidant molecules in humans, UA has the valid ability to clear out peroxynitrite, nitric oxide, and hydroxyl radicals; hence, it can prevent protein nitration and lipid peroxidation (38, 39). Studies in animal models have shown that administration of UA or soluble UA analogs that retain the antioxidant properties of UA protects the brain against ischemic injury (40–42). However, once it exceeds the normal range, SUA would impact multiple systems, which in turn lead directly or indirectly to stroke. Possible mechanisms have been reported that elevated UA level was associated with carotid intima media thickness, as reported by the latest meta-analysis; high UA was related to carotid intima thickening (43); and the same trend was found in proximal extracranial artery stenosis (44). Meanwhile, it was demonstrated that elevated UA promoted atherosclerotic progression by increasing production of free radicals and facilitating low-density lipoprotein cholesterol (LDL-C) oxidation and lipid peroxidation (45). In addition, high levels of UA increased vascular endothelial dysfunction (46) and vascular smooth muscle cell proliferation, which could lead to preglomerular vascular disease and high blood pressure (47, 48). Potential mechanisms have also been reported that elevated UA level was involved in microvascular injury (47), increasing platelet aggregation and thrombus formation (49). Studies had revealed that UA could increase inflammatory cytokines such as C-reactive protein, interleukin 6 (IL-6), and tumor necrosis factor α (TNF-α) (50). Simultaneously, clinical studies also suggested that high SUA levels increased the risk of total mortality and cardiovascular and cerebrovascular diseases. In Italy, a national multicenter retrospective cohort study (51) assessed that all-cause mortality was substantially increased when the UA levels were above 4.7 mg/dl (95% CI: 4.3–5.1 mg/dl), and the risk of cardiovascular mortality (CVM) ascended while the value of SUA is over 5.6 mg/dl (95% CI: 4.99–6.21 mg/dl). These findings from experimental, epidemiological, and clinical studies of UA suggested that elevated SUA could be associated with vascular diseases and clarified the important role played by SUA levels in illustrating the possible pathophysiological association with hypertension, atherosclerosis, and stoke.

In our study, we took ischemic stroke and hemorrhagic stroke as the main subtypes, and we found elevated SUA levels have similar adverse effects on the development of stroke in these two subtypes. Evidence showed that ischemic stroke and hemorrhagic stroke both cause local hypoxia that damage brain tissue, and they could be converted to each other. There was a high risk of hemorrhagic transformation during the treatment of ischemic stroke (52). However, the study presented that significant differences existed in body composition between hemorrhagic and ischemic stroke in humans, and individuals with ischemic stroke had significantly worse body composition (53). Further exploration of the molecular mechanisms of SUA and different types of stroke is noteworthy.

Sex differences in the association of elevated SUA with stroke-related risk factors were found in our study. Statistically, females have a higher risk of experiencing a stroke-related fatality than males. Meanwhile, a J-shaped trend between the ascending SUA levels and higher risk of stroke for men and a liner trend for women had been explored. It is universally acknowledged that stroke is a sexually dimorphic disease. For one reason, females have a longer average lifespan, which increases the odds that they will have a stroke. Besides, females suffer greater susceptibility to depression and anxiety and often report higher levels of stress than males do (54–56). Other unique risk factors that females are facing, such as gestational hypertension and climacteric syndrome, may also cause the difference. To conclude, differences in vascular biology, immunity, coagulation, hormonal profiles, lifestyle factors, and societal roles seem to contribute (57).

Some limitations for the present meta-analysis should be acknowledged. Firstly, we were unable to carry out further subgroup comparison of hemorrhagic stroke because the corresponding data were not available in the original articles. The mechanisms and risk factors for subarachnoid hemorrhage and intracerebral hemorrhage are different in important ways, as are treatment and outcomes (58). More clinical and mechanistic studies deserve further research. Secondly, even though the errors of dose–response analysis are unavoidable in secondary analysis, the overall *J*-shaped trend is worthy of our attention in the relationship of SUA and risk of stroke in this meta-analysis. Thirdly, although a large panel of subgroup analyses were undertaken to account for possible sources of heterogeneity, significance still persisted in some subgroups, limiting the interpretation of pooled effect-size estimates. Finally, similar to any observational studies, a causal relationship could not be fully established.

## Conclusions

Our study found that elevated SUA is a significant risk factor for adult stroke, both for ischemic stroke and hemorrhagic stroke, especially in females. Our dose–response research revealed a *J*-shaped trend between the ascending SUA levels and the higher risk of suffering from stroke. Moreover, high SUA levels are associated with an increased risk of fatal stroke. Further investigations on the molecular mechanisms linking SUA to adult stroke are also warranted.

## Data Availability Statement

The original contributions presented in the study are included in the article/Supplementary Material, further inquiries can be directed to the corresponding author/s.

## Author Contributions

TQ reviewed the articles and wrote the manuscript. HW helped with the article review. WP was the editor of the manuscript and helped with the preliminary qualification. All authors contributed to the article and approved the submitted version.

## Conflict of Interest

The authors declare that the research was conducted in the absence of any commercial or financial relationships that could be construed as a potential conflict of interest.

## Publisher's Note

All claims expressed in this article are solely those of the authors and do not necessarily represent those of their affiliated organizations, or those of the publisher, the editors and the reviewers. Any product that may be evaluated in this article, or claim that may be made by its manufacturer, is not guaranteed or endorsed by the publisher.
