# Supplementary material for: The Relationship Between Elevated Serum Uric Acid and Risk of Stroke in Adult: An Updated and Dose–Response Meta-Analysis
Source: Front Neurol. 2021 Aug 10;12:674398. doi: 10.3389/fneur.2021.674398 (PMC8435906; doi:10.3389/fneur.2021.674398)
Supplement: Supplementary file 1 [file Table_1.docx]

**Supplementary table1. Baseline characters of the associations between UA levels and the risk of having stroke.**

| **Author** | **Year** | **Location** | **Study design** | **Uric acid Assessment** | **Outcome Assessment** | **Adjustment** |
| --- | --- | --- | --- | --- | --- | --- |
| Sakata | 2001 | Japan | Prospective cohort | Colorimetric phosphotungstic acid procedure | ICD-9 | Age, body mass index, systolic blood pressure, use of antihypertensive agents, serum total cholesterol level, serum creatinine level, serum glucose level, smoking status, alcohol intake, and left ventricular hypertrophy. |
| Chien | 2005 | China | Prospective cohort | Enzymatic with commercial kits | Preliminary diagnoses, death certificates | Age, systolic blood pressure, body mass index, diabetes, LDL-C, HDL-C, smoking, drinking, electrocardiographic left ventricular hypertrophy and atrial fibrillation history. |
| Bos | 2006 | Netherlands | Prospective cohort | Kone Diagnostica reagent kit | Hospital records | Age |
| Gerber | 2006 | Israel | Prospective cohort | Fister’s adaptation of colorimetric method | ICD-9 | Age, body mass index, systolic blood pressure, diabetes, serum cholesterol, smoking, and left ventricular hypertrophy on electrocardiogram. |
| Hozawa | 2006 | USA | Prospective cohort | Uricase method | ICD-9 | Age, sex, race, education, systolic blood pressure, diabetes mellitus, anti-hypertensive medication, cigarette smoking status, ethanol intake, serum albumin, von Willebrand factor, body mass index, waist-to-hip ratio, and low HDL-C. |
| Strasak1 | 2008 | Austria | Prospective cohort | Enzymatic method | ICD-9 and IDC-10 | Age, body mass index, systolic and diastolic blood pressure, total cholesterol, triglycerides, gamma-glutamyl transferase, glucose, smoking status, occupational status and year of examination. |
| Strasak2 | 2008 | Austria | Prospective cohort | Enzymatic method | ICD-9 and IDC-10 | Age, body mass index, systolic and diastolic blood pressure, total cholesterol, triglycerides, GGT, glucose, smoking status, and year of examination. |
| Holme | 2009 | Sweden | Prospective cohort | Enzymatic uricase method | ICD-7, ICD-8, ICD-9, ICD-10 | Age, TC, TG, hypertension and DM. |
| Storhaug | 2013 | Norway | Prospective cohort | Enzymatic colorimetric test | Hospital or out-hospital records | Age, BMI, SBP, DBP, HDL-C, TC, renal factors, use of diuretics and antihypertensive medication, current smoking and physical activity. |
| Zhang | 2016 | Japan | Prospective cohort | Colorimetric phosphotungstic acid | ICD-9 and ICD 10 | Age, body mass index, smoking status, ethanol intake, systolic blood pressure and total cholesterol. |
| Shi | 2017 | China | Prospective cohort | NA | Endpoint adjudication committee | Age, study centers, body mass index, systolic blood pressure, total cholesterol, triglycerides, glucose, eGFR, homocysteine, smoking status, and alcohol consumption at baseline and systolic blood pressure during treatment period. |
| Tu | 2019 | China | Prospective cohort | NA | IDC-10 | Age, gender, smoking and drinking habits, along with baseline BMI, systolic and diastolic BP and baseline eGFR, triglycerides, total cholesterol, HDL, and LDL . |
| Chaudhary | 2020 | USA | case-cohort design | Roche colorimetric assay | Medical records | Age, race, interaction between age and race, hypertension, aTRH, smoking, diabetes mellitus, atrial fibrillation, left ventricular hypertrophy, coronary artery disease, aspirin, lipid-lowering medications, warfarin, estimated glomerular filtration rate, and high-density lipoprotein level. |
| Li | 2020 | Japan | Prospective cohort | Phosphotungstic acid method | Medical records | Age, community, body mass index, cigarette smoking status, alcohol intake status, systolic blood pressure, atrial fibrillation, serum total cholesterol, serum triglycerides, estimated glomerular filtration rate, diabetes mellitus, antihypertensive medication use, and in women, menopausal status. |
| Norvik | 2017 | Norway | Prospective cohort | Enzymatic colorimetric test | Medical records | Sex, age, BMI, mean systolic blood pressure, mean diastolic blood pressure, total cholesterol, triglycerides, eGFR, HbA1c, current smoking, physical activity, use of antihypertensive medication, uric acid, E/A ratio |
| Chen | 2009 | China | Prospective cohort | NA | IDC-9 | Age, sex, body mass index, cholesterol, triglycerides, diabetes, hypertension, heavy cigarette smoking, and frequent alcohol consumption, and were stratified by sex. |
| Chen | 2011 | China | Prospective cohort | NA | Hospital records | Age, gender, pulse pressure, diabetes. |
| Koton | 2008 | UK | Prospective cohort | Gilford Impact 400E analyzer | Brain imaging or post-mortem data | Age, sex, SBP, total cholesterol, smoking, diabetes, antihypertensive treatment, previous MI or angina and BMI. |
| Lehto | 1998 | Finland | Prospective cohort | Enzymatic calorimetric method | IDC-9 | Body mass index, total triglycerides, HDL-C, plasma glucose, previous history of stroke, use of diuretics, and duration of diabetes. |

**Abbreviations:** LDL-C, low-density lipoprotein cholesterol; HDL-C, high-density lipoprotein cholesterol; GGT, gamma-glutamyl transpeptidase; TC, total cholesterol; TG, triglyceride; DM, diabetes mellitus; BMI, Body mass index; SBP, systolic blood pressure; DBP, diastolic blood pressure; eGFR, estimated glomerular filtration rate; aTRH, apparent treatment-resistant hypertension; E/A ratio, the ratio of peak early Doppler mitral flow velocity (E-wave) to peak late Doppler mitral flow velocity (A-wave); HbA1c, Glycosylated Hemoglobin; MI, [myocardial infarction](javascript:;); IDC, international Classification of Diseases; UK, the united kingdom; USA, the united states; UA, uric acid; NA, not available.
